# Bony Tumor of the Scrotum

**Published:** 1858-06

**Authors:** 


*Bony Tumor of the Scrotum* By JOHN G. KERR, M. D., late of Canton, China.

A man twenty-eight years old, of good constitution, and enjoying good health, was admitted into the Ophthalmic Hospital at Canton, China, in September, 1856.

Twenty months before the time of admission, a tumor began to grow in the patient's scrotum. It steadily increased, and had now attained such a size as to be very inconvenient, and to interfere with his occupation, which was that of a laborer in the country. The tumor was about as large as an infant's head, very hard and dense, and seemed to the touch almost like a mass of stone or wood inclosed within the skin. The skin was healthy, and freely movable over all parts of the tumor.

At my request the operation for its removal was performed by Dr. Geo. B. Newton, Assistant Surgeon of H. B. M. brig "Bittern," who had rendered me important assistance in several operations. The patient was placed under the influence of chloroform, and the tumor skilfully dissected away. The left testicle was carried down before the tumor, and was removed with it. Both testicles were healthy, excepting a slight hydrocele.

The weight of the tumor was five pounds, and was found to consist of numerous cartilaginous lobes of various sizes, densely compacted together with cellular tissue, in which large quantities of bone were deposited. When macerated and cleaned, it presented somewhat the appearance of a coral formation, springing from an irregular semicircular base. Numerous spiculæ of bone were scattered througout the tumor where apparently separate points of ossification had been established in the different lobules. So numerous were these spiculæ that the knife could scarcely be put into the tumor without touching them.

A part of the bone has a compact outer plate, but the greater portion is spongy and cellular, and composed of radiating spiculæ attached to a firmer base. The process of ossification was interrupted by the operation, and as the tumor had not ceased growing, it is likely that the anomalous formation would have continued to enlarge indefinitely if it had not been removed.—*North American Medico-Chirurgical Review.*

---

*Ts'ün T'ai San Lun.* "*A new Description of the Entire Boky.*" By Benjamin Hobson, M.B. Published at the "Wai Oi" Hospital, Canton, China.

This book, whose uncouth title heads this article is an octavo volume of 152 pages, printed in the Chinese character. It is an epitome of the principal facts of anatomy and physiology as they are known to the profession in Europe and America. Beginning at osteology, the various structures of the system and the functions of the organs are described, and practical remarks on medicine and surgery are introduced. The treatise concludes with an account of the development of the fœtus in utero, and some of the accidents of parturition. It is illustrated with numerous wood cuts—copies of those in common use in modern works on anatomy and physiology.

It is not easy to appreciate the difficulty of transferring a science into a new language where a nomenclature has to be invented and words applied to the description of objects hitherto unknown. This was the labor undertaken by Dr. Hobson, for his work is the first attempt to introduce to the inhabitants of the celestial empire a true account of the structure and functions of the human system. That he has succeeded in his effort to a very encouraging extent is shown by the estimation in which his book is held by intelligent natives. A large edition has been published by a wealthy native merchant, with an introduction acknowledging its superiority, yet taking exceptions to some parts as altogether chimerical.

The father of Yeh, the present Governor General of Canton, published a very fine edition of the plates. They make eight sheets, a foot wide by three long, and are a complete set of anatomical plates. A higher compliment could not be paid to any book, especially one of foreign origin, for it must be remembered that educated natives look with profound contempt upon anything which comes from the "barbarian or red-haired devils."

The benefit which Dr. Hobson has attempted to confer upon 300,000,000 of the human family can only be estimated by considering that they know nothing of anatomy and physiology, and that not even an attempt is made at the practice of surgery. Space does not permit us to enlarge upon the general state of the profession and the practice of medicine among our antipodes. Of this more anon.—*North American Med. Chirg. Review.*

---

*Insanity and Crime.*—The Courts of Law constantly afford proofs that among young children there is a form of insanity which, beginning in what might be termed mischief-disease, ends in offences against human life of the most fearful kind. The newspapers, this week, afford some examples of offences of this kind, committed apparently without the slightest provocation. The first and most extraordinary instance we find quoted in a continental journal. A little boy, not more than nine years of age, having enticed five of his companions into a large box, shut the cover down, and sat cross-legged upon it, seeming to enjoy the groans of his expiring playmates. After he had discovered by inspection that they were all dead, he proceeded to a field and flew his kite, apparently without one pang of remorse for the dreadful murder he had just committed! In the Lambeth Police Court, on Tuesday, an inquiry took place respecting a similar unpremeditated and unmean ing attack upon human life, made by a lad named James Reynolds, sixteen years of age. It appears that, a fortnight since, he was seen, without the slightest provscation, to take up a child of seven years of age, and throw it into the Surrey Canal; and then, as if to make the crime more marked, he went to the person who had charge of the little one, and informed her that it was drowned. The child was fortunately rescued; but the act was completed as far as lay in the power of the lad. We do not know what course will be taken with the perpetrator of the five-fold homicide, for we cannot call it murder; but in the latter case the lad was fined £5, and, in default, two months hard labor! Now, there can be no matter of doubt that both offenders were laboring under a certain form of madness; and to fine, or to punish them by a slight term of incarceration, is absurd. They should be removed permanently from society; if not, we may expect to hear of a repetition of those fearful acts. It is one of the maxims of law, that it is necessary to prove some motive for the perpetration of an extraordinary offence; but the insane perform the most extraordinary acts without the slightest shade of motive, speaking in a natural sense; and in the latter of these cases we have an apt example of the errors lawyers may commit, unenlightened as they are by the truths of psychological medicine.—*British Medical Journal,* from *The Medical News and Library.*

---

*Perineuron, a New Element in Nerves.*—Dr. C. Robin has recently described a new tissue altogether distinct from the neurolemma investing nervous matter. Its characters are

1. Its situation around the primitive bundles of nerve-tubes and sometimes around the tubes themselves.

2. Its tabular form and its ramifications accompanying the ramifications of the nerves.

3. Its chemical reäctions with various tests.

4. A thin, transparent wall, finely granulated, often presenting longitudinal striæ and nuclei, more numerous in the small tubes than in the large.

The perineuron is to the nerve-tubes what the saroclemma is to the muscular fibres, the immediate investment of the proper nerve-tissue. It is found in all the nerves of animal life, including the vagus, in all their length from the ganglia for the sensitive nerves, and for those which do not pass through ganglia, from the cerebro-spinal axis. The three nerves of special sense are the only exceptions to this, they not having the perineuron. In the sympathetic nerve, this envelop exists only where there are white bundler or ramifications, and not among the gray or gelatiniform bundles.

In large nerves, the tubes of the perineuron are large (two to five-tenths of a millimetre,) in ramifications they become smaller, containing then only two or three nerve-tubes, and sometimes only one. The capillaries do not go inside these tubes.—*American Journal of Dental Science.*

---

*Progress of Invention.*—Much ingenuity is continually brought to bear upon the construction of instruments employed in medical and surgical practice. All the departments of our art are largely indebted to cutlers and others engaged in the fabrication of the various appliances so necessary to safe and expeditious operations and dressings. There is a new otoscope, or speculum auris, invented by Mr. Tiemann, of New York, which seems to realize the wishes of aurists, in the completeness with which it allows of a view of the tympanum. Dr. Bethune, who showed the instrument at the last meeting of the Medical Improvement Society, states that the vessels of the tympanum can be distinctly seen by it, and that the membrane itself is enlarged, by the lens through which it is viewed, to about the size of a dime. By means of reflected light, all the difficulties so long experienced in getting sufficient rays to fall upon the tympanum are avoided. The polished funnel which receives the light is directed upward, so as to throw it upon a small mirror within, whence it is reflected inward powerfully. The observer looks straight forward, as toward any object-glass. Several gentlemen tested the magnifying power of the instrument.

What with the double stethoscope, the ophthalmoscope, the improved microscope, and the new otoscope, the *scope* of the profession is likely to be increased beyond anything that ancient *horoscopes* could have divined!—*Boston Medical and Surgical Journal.*

*Rupture of the Trachea by a Fall.*—Dr. Atlee, of Lancaster, Pa., reports a case in the January number of the American Journal, of death produced by a fall, the subject, a lad of four years, stricking his neck against a scraper at the side of the door. He says: "I reached the house not more than five minutes after the injury had been received, and the child was then seated upon his mother's lap, his head resting against her arm, and breathing naturally, or nearly so. There was some blueness of the lip, but this soon passed off; on his countenance there was not much appearance of distress. Where the neck had come in violent contact with the scraper, there was not the slightest mark upon the skin. I was just about to congratulate the family upon the slightness of the injury, when the child, struggling to free himself from his mother's arms, threw himself violently backward. He at once became enormously swollen, and in a moment was dead. The cause of the swelling was evidently the entrance of air into the cellular tissue, and it extended over the head, the neck, the trunk, and the upper extremities to the ends of the fingers. At the sternum, the finger, before reaching the bone, penetrated fully an inch." There was no post mortem examination.—*Cincinnati Lancet and Observer.*

---

*Syphilization*, which is now a growing innovation, receives a support from India, as we learn from a letter from M. Guepin, of Nantes, to Dr. Caffé.

Syphilization is practised in certain parts of India apon a great scale, as a preventive as well as a curative means. The prostitutes of India often offer to syphilize our sailors, but they always refuse, unwilling to submit to any means not employed in Europe. The marine officer who gave me this information had the curiosity to see some of the women who offered to syphilize his sailors, and did not observe apon their bodies any disease of the skin, nor any cicatrix of a bubo. This fact is the more important, because he did not know that syphilization had been re-invented in France, practised with success in both Turin and Christiana, and also for the reason that syphilitic diseases contracted in India are excessively severe; those afflicted with it very often die, or are cured very slowly, after being subjected to the ablest, the most methodical, and energetic treatment.—*American Med. Monthly.*

